# Cortical signatures of vicarious tactile experience in four-month-old infants

**DOI:** 10.1016/j.dcn.2017.09.003

**Published:** 2017-09-14

**Authors:** Silvia Rigato, Michael J. Banissy, Aleksandra Romanska, Rhiannon Thomas, José van Velzen, Andrew J. Bremner

**Affiliations:** a Centre for Brain Science, Department of Psychology, University of Essex, Colchester, CO4 3SQ, UK; b Sensorimotor Development Research Unit, Department of Psychology, Goldsmiths, University of London, London, SE14 6NW, UK

**Keywords:** Multisensory development, Touch, Infancy, Empathic sensing, Somatosensory evoked potentials, Tactile perception, Social perceptual development

## Abstract

The human brain recruits similar brain regions when a state is experienced (e.g., touch, pain, actions) and when that state is passively observed in other individuals. In adults, seeing other people being touched activates similar brain areas as when we experience touch ourselves. Here we show that already by four months of age, cortical responses to tactile stimulation are modulated by visual information specifying another person being touched. We recorded somatosensory evoked potentials (SEPs) in 4-month-old infants while they were presented with brief vibrotactile stimuli to the hands. At the same time that the tactile stimuli were presented the infants observed another person’s hand being touched by a soft paintbrush or approached by the paintbrush which then touched the surface next to their hand. A prominent positive peak in SEPs contralateral to the site of tactile stimulation around 130 ms after the tactile stimulus onset was of a significantly larger amplitude for the “Surface” trials than for the “Hand” trials. These findings indicate that, even at four months of age, somatosensory cortex is not only involved in the personal experience of touch but can also be vicariously recruited by seeing other people being touched.

## Highlights

• Somatosensory ERPs are modulated by seeing other people being in 4-month-olds.
• Visual touches modulate the early feedforward stages of somatosensory processing.
• A demonstration of the developmental origins of sensory empathy.
• The primacy of social perception in early life extends to the tactile sense.

## Graphical abstract

**Figure:**
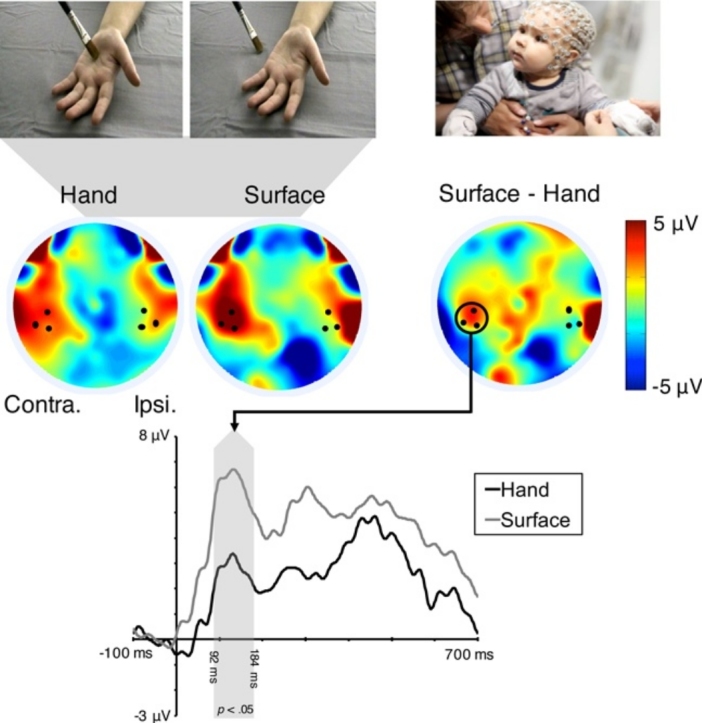

## 1 Introduction

Research tracing the early origins of social perception has focused on infants’ processing of visual, auditory, and audiovisual social events (see Bahrick and Lickliter, 2014, Pascalis and Kelly, 2009). And yet *touch* is our earliest developing sense and likely plays an important role in early social perception (Humphrey, 1964; see Bremner and Spence, 2017). When visual and auditory systems are still maturing in function and anatomy, touch gives clear indications concerning the proximity of a caregiver. Touch is important in the early establishment of reciprocal interactions and attachment between infant and caregiver (e.g., Harlow and Zimmerman, 1959) and plays an important role in affect regulation (e.g., the analgesic effect of touch during heel lance procedures with neonates; Gray et al., 2000). Nonetheless, the developmental origins of our ability to perceive social tactile events and combine those with a wider social sensory context have gone largely untreated until very recently (see Fairhurst et al., 2014, Filippetti et al., 2013).

Given the foundational role of touch in social perception, an important question concerns how tactile perception of the social world becomes integrated with social responses and behaviours mediated by the other senses. As adults, we have a transparent appreciation of the meaning of tactile experiences when we observe those happening to others. This “vicarious mapping” is a part of our capacity to share the experiences of others (i.e. empathy), a critical aspect of human behavior (Bird and Viding, 2014). One characteristic of the mature human brain that lends itself to this vicarious sensory empathy is the recruitment of similar brain regions when a state is experienced and when that state is observed in others (Keysers and Gazzola, 2009). For instance, seeing other people being touched, or touching objects, activates similar brain areas as when we experience touch ourselves (Blakemore et al., 2005, Ebisch et al., 2008, Ishida et al., 2010, Keysers et al., 2004, Meyer et al., 2011, Pihko et al., 2010, Schaefer et al., 2009). Particularly pertinent for this investigation, effects of observed touch and painful touch on somatosensory processing have also been observed in somatosensory evoked potentials (SEPs; Bufalari et al., 2007, Martínez-Jauand et al., 2012). But while we know much about brain regions involved in vicarious mapping of tactile experiences in human adults (e.g., see Keysers et al., 2010), when and how this develops in human infancy remains largely unaddressed (Gillmeister et al., 2017). Here we report an investigation of the developmental origins of vicarious tactile mapping and empathy in human infancy. We examined whether 4-month-old infants’ somatosensory evoked potentials (SEPs) measured in scalp EEG were modulated by visual observations of touches to another person’s hand.

Somatosensory functions at and before birth likely play a foundational role in body perception in both solipsistic and social contexts. Cortical responses to tactile and painful stimulation have been observed in newborns (Bartocci et al., 2006, Goksan et al., 2015, Pihko et al., 2004), and preterm newborns from the onset of thalamic connections to somatosensory cortex (∼24 week gestational age; Hrbek et al., 1973, Milh et al., 2007, Nevalainen et al., 2014). Some topographic mapping of the body in somatosensory cortex has been observed in preterm infants (Milh et al., 2007) and in later infancy (Saby et al., 2015), and spatial mapping of touch is also seen in early behaviours (e.g., Dumont et al., 2017, Kisilevsky and Muir, 1984). Nonetheless, a number of studies now demonstrate some significant postnatal changes in the ways infants perceive touch (Bremner, 2016). An ability to refer touches to locations in visual external space (e.g., across change in limb posture) develops significantly (Begum Ali et al., 2015, Bremner et al., 2008, Rigato et al., 2014) as a result of visual/multisensory experience in the first months of life (Azañón et al., 2017, Ley et al., 2013). Visual-tactile interactions underlying our perception of hand/body position continue to develop until late in childhood (Bremner et al., 2013, Cowie et al., 2016, Cowie et al., 2017, Nava et al., 2017).

If visual-tactile interactions are constrained across domains in early life, we might predict that visually-observed touches to other people should not influence somatosensory processing. However, there are good reasons to doubt this. Firstly, many studies show that crossmodal interactions influence infant behaviour from early infancy (e.g., Bahrick and Lickliter, 2014, Lewkowicz et al., 2010, Slater et al., 1999), and a number of studies have shown visual-haptic transfer in newborns (Sann and Streri, 2007, Meltzoff and Borton, 1979; although see Maurer et al., 1999, and Gori et al., 2008). Secondly, the kinds of multisensory inputs which might mediate learning about relations between visually-observed touch events and somatosensory inputs are probably available early: Infants have rich opportunities to observe body parts (both their own and others’) in the visual field (Fausey et al., 2016), and instances where this happens are likely to be highly correlated with somatosensory stimulation. In light of these observations, we predicted that we should observe vicarious mapping of tactile sensation in early infancy.

We investigated vicarious mapping of touch in a group of 4-month-old infants by probing whether somatosensory processing is modulated by visual observations of touches to another person’s hand. Following Bufalari et al.’s (2007) study with adults, we showed infants “touch events” on a video screen (∼3.5 s duration), in which a paintbrush either touched a stationary hand or the table surface next to the hand. Synchronously with the visually-specified tactile contact, we presented vibrotactile stimuli to one of the infants’ hands (selected randomly) for 200 ms whilst recording scalp EEG. We examined whether somatosensory evoked potentials (SEPs) were modulated by seeing a hand being touched as opposed to seeing a surface being touched. In adults the amplitude of the contralateral P45 of the SEP is suppressed when a simultaneous visually presented touch is shown to the hand, as compared to a touch to a surface (Bufalari et al., 2007). We focussed on modulations of early SEP components at central scalp sites. Given the age group, no predictions were made concerning the direction of the modulation (DeBoer et al., 2007).

## 2 Methods

### 2.1 Participants

Fifteen four-month-olds (6 males), aged between 104 and 150 days (mean age 129 days) participated in the experiment. An additional 7 infants began participating but were excluded from the analyses because of fussy behaviour. Informed consent was obtained from the parents. The testing took place only if the infant was awake and in an alert state. Ethical approval was gained from the Research Ethics Committee of Goldsmiths, University of London. The studies conform with The Code of Ethics of the World Medical Association (Declaration of Helsinki; British Medical Journal, 18 July 1964).

### 2.2 Design

The infants were presented with a series of trials in which 200 ms vibrotactile stimuli were delivered to one of their hands (whether left or right hand was randomised across trials). We measured somatosensory evoked potentials (SEPs) in the scalp EEG (see also Rigato et al., 2014). At the same time as the vibrotactile stimuli, the infants were shown touching events on a video screen (lasting approx. 3.5 s), in which a paintbrush either touched a stationary hand (Hand trials), or the table surface next to the hand (Surface trials). The visual moments of touch (of either the hand or the table surface) were timed to coincide with the onset of the vibrotactile stimuli applied to the infants’ hands. The order in which the Hand and Surface video conditions were presented was randomised. Vicarious mapping of visually perceived touches on another person’s hand to the touches felt on the infants’ own hands was operationalised as the wave of the difference between SEPs recorded on Hand and Surface trials. Infants were presented with a maximum of 100 experimental trials (i.e., a maximum of 50 trials for each of the Hand and Surface conditions).

### 2.3 Stimuli and apparatus

**Fig. 1:**
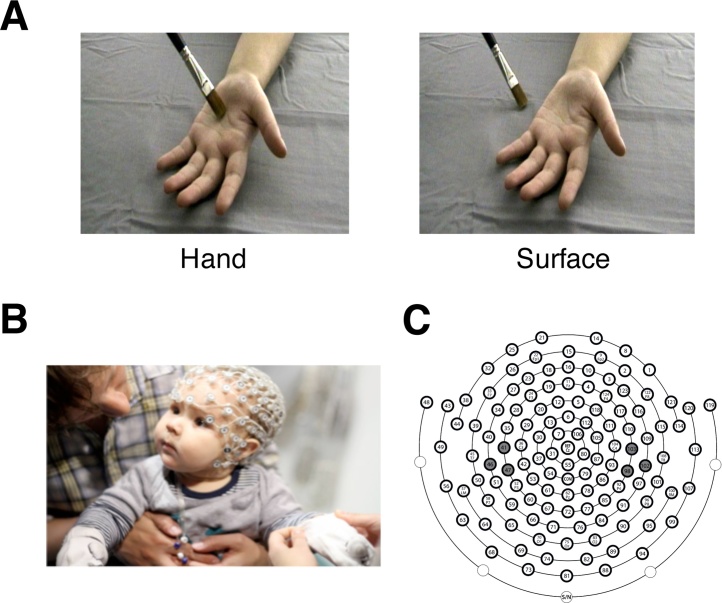
Experimental procedures. (A) The visual presentations of Hand and Surface conditions. (B) A picture of an infant receiving vibrotactile stimuli to the hands whilst SEPs are recorded via scalp electrodes. (C) The layout of the Hydrocel Geodesic Sensor Net used in the experiment. The electrodes which were selected for analysis are shaded (Left hemisphere/CP3: 41, 46, 47; Right hemisphere/CP4: 98, 102, 103).

All of the infants were tested in a dimly lit room, seated on their parent’s lap with their forearms held by the parent and resting on a small table. EEG was recorded throughout the experimental session. The 200 ms vibrotactile stimuli were presented via in house custom-built voice coil tactile stimulators (tactors), driven by a 220 Hz sine wave. One tactor was placed in each hand. The tactors were fixed to the infants’ palms with elastic straps, one in each hand. The infants’ hands and the tactors were then covered by small cotton mittens (see Fig. 1). In order to mask the noise of the tactors, white noise was played ambiently in the room throughout the experiment.

The visual tactile events were presented as video clips on a 21” screen at 90 cm distance from the infant’s head. These video clips presented a left hand from a third-person perspective, as if another person was facing the infant, in two different conditions (see Fig. 1): Hand and Surface (described above). In order to maintain the infants’ attention, we also included 50 randomly interleaved trials of the same duration as the experimental trials (3.5 s). In these “filler” trials, dynamic cartoon images were presented at the same time as the vibrotactile stimuli.

### 2.4 Procedure

Once the tactors were placed in and attached to the infant’s hands, the parent was instructed to, as far as was possible, hold the wrists of the infant approximately 10 cm apart for the duration of the experiment. Two video cameras were mounted in the experimental room to monitor the infants’ eye- and head-movements, and to ensure that they adopted and maintained the given hand position. When the infants’ hands and eyes moved out of position, testing was paused until the posture was readopted and visual attention reattained. The orders in which the video conditions were shown and the vibrotactile stimuli were presented across hands were randomised. Each trial lasted about 3.5 s and was preceded by a 1 s screen with a central fixation stimulus (a small black and white pattern).

### 2.5 EEG recording and analyses

Brain electrical activity was recorded continuously by using a Hydrocel Geodesic Sensor Net, consisting of 128 silver–silver chloride electrodes evenly distributed across the scalp. The vertex served as the reference. The electrical potential was amplified with 0.1–100 Hz band-pass, digitized at a 500 Hz sampling rate, and stored on a computer disk for off-line analysis. The data were analysed using NetStation 4.4 analysis software (Electrical Geodesic Inc.). Continuous EEG data were low-pass filtered at 30 Hz using digital elliptical filtering, and segmented in epochs from 100 ms before until 700 ms after stimulus onset. Segments with eye-movements and blinks were detected visually and rejected from further analysis. Artifact-free data were then baseline-corrected to the average amplitude of the 100 ms interval preceding stimulus onset, and re-referenced to the average potential over the scalp. Finally, individual and grand averages were calculated. The average numbers of trials included in the analyses were 18.3 for the Surface condition, and 17.9 for the Hand condition. Although this number of artefact-free ERP trials per condition is low in comparison to numbers typically gathered for adult participants it is in line with norms for ERP studies with young infants (see De Boer et al., 2007, who explain that fewer trials are typically needed in infant participants due to higher resilience to movement artefacts, likely due in turn to physiological factors such as thinner skulls and less dense cell packing in brain tissue).

Statistical analyses of the ERP data focused on sites close to somatosensory areas contralateral and ipsilateral to the stimulated hand. We also focused on the early stages of somatosensory processing (0–200 ms) because prior studies have observed: i) visual effects on the early stages of somatosensory processing in infants (Rigato et al., 2014), and ii) effects of visually observed touches on the early stages of somatosensory processing in adults (Bufalari et al., 2007). We identified scalp regions surrounding CP3 and CP4 of the 10–20 system, based on the presence of hotspots prior to 200 ms in the topographic maps. Next, electrodes within these areas were visually inspected in order to identify a representative sample of electrodes, symmetrical across the hemispheres, that showed the most pronounced SEP components as well as low levels of between-participant variability. In consequence, the analyses focussed on the following centroparietal electrodes: 41, 46, 47 (left hemisphere); 98, 102, 103 (right hemisphere) (see Fig. 1).

The analyses focussed on the most prominent feature of the grand-averaged SEP, which was a positive peak around 130 ms after onset of the tactile stimulus. The latencies of the peak amplitude were determined for each individual participant by visual inspection, and the time window was then chosen to include the temporal spread of peaks across participants. This resulted in a window between 0 and 200 ms. This peak amplitude was compared across conditions (Hand and Surface) and hemispheres (Contralateral and Ipsilateral) with a sample point by sample-point analyses. We used a Monte Carlo simulation method (based on Guthrie and Buchwald, 1991) in which we were able to trace the time course of statistically reliable modulations of the SEPs by Condition (Hand/Surface) on a sample-point basis (intervals every 2 ms) across difference waveforms for each hemisphere.

The Monte Carlo simulation method (Guthrie and Buchwald, 1991) began by averaging the first order autocorrelation present in the real difference waveforms across the temporal window noted above. Next, 1000 datasets of randomly generated waveforms were simulated, each waveform having zero mean and unit variance at each time point, but having the same level of autocorrelation as seen on average in the observed data. Each simulated dataset also had the same number of participants and time-samples as in the real data. Two-tailed one sample *t*-tests (vs. zero; alpha = 0.05, uncorrected) were applied to the simulated data at each time point, recording significant vs. non-significant outcomes. In each of the 1000 simulations the longest sequence of consecutive significant *t*-test outcomes was computed. The 95th percentile of that simulated distribution of “longest sequence lengths” was then used to determine a significant difference waveform in the real data; specifically, we noted any sequences of significant *t*-tests in our real data which exceeded this 95th percentile value. This method thus avoids the difficulties associated with multiple comparisons and preserves the type 1 error rate at 0.05 for each difference waveform analysed.

## 3 Results

**Fig. 2:**
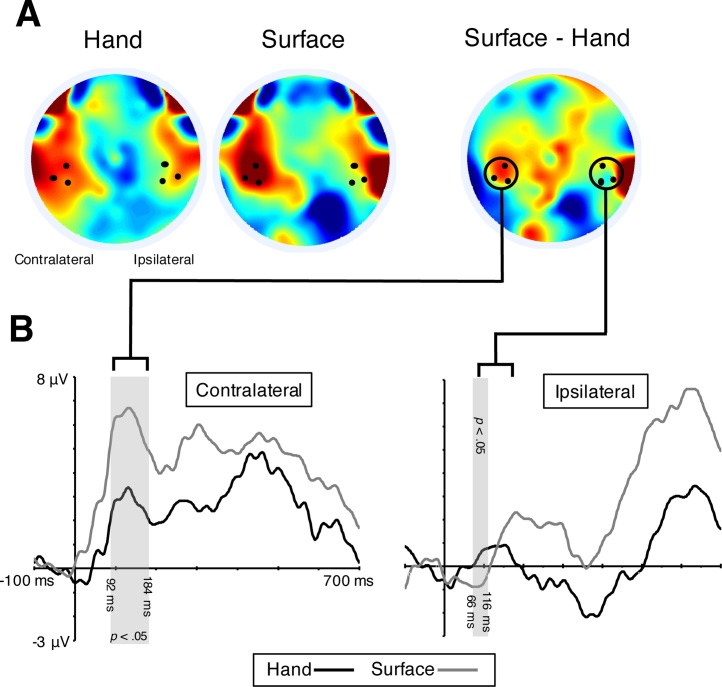
Visually presented touches on another person’s hand modulated somatosensory evoked potentials (SEPs) in 4-month-old infants. (A) Grand average topographical representations of the voltage distribution over the scalp in the Hand and Surface conditions between 92 and 184 ms after stimulus onset (the period during which the Surface-Hand contrast was significant at contralateral sites), with a Surface – Hand difference map to the right. (B) Grand average SEPs at contralateral (left) and ipsilateral (right) centroparietal sites (CP3/CP4). The statistically reliable effects of condition on the SEP amplitude at contralateral (between 92 and 184 ms) and ipsilateral sites (between 66 and 116 ms) are indicated by the grey shading.

We used the Monte Carlo simulation method described above to make comparisons of SEP amplitude in the Hand and Surface conditions during the positive going component in the SEP centred around 130 ms. Firstly, a comparison in the Contralateral hemisphere revealed a statistically reliable effect of Condition on SEP amplitude from 92 to 184 ms (Hand: *M* = 3.06 μV; Surface: *M* = 5.80 μV; see Fig. 2). The simulation, which was run with a first order autocorrelation of 0.99 at lag 1, identified any sequence of consecutive significant *t*-tests longer than 44 ms to be reliable. Thus, the sight of a hand being touched appears to result in a reduction of somatosensory processing at contralateral sites during the feedforward stages of somatosensory processing (from 92 ms).

One possibility which we wanted to exclude was that the Hand condition may have resulted in a fairly unspecific suppression across larger areas of the brain, rather than a specific modulation of somatosensory processing. In order to determine whether the effect of condition was specific to the contralateral hemisphere we also conducted a comparison of the Condition contrast waveforms across Hemispheres. This confirmed that there was a significant Condition x Hemisphere interaction from 66 to 160 ms. The simulation, which was run with a first order autocorrelation of 0.98 at lag 1, identified any sequence of consecutive significant *t*-tests longer than 38 ms to be reliable. We followed up these planned tests with an exploratory comparison of the SEP across Conditions in the Ipsilateral hemisphere. Interestingly, this test also demonstrated a significant effect of Condition between 66 and 166 ms, but in the opposite direction to the effect seen contralaterally (Hand: *M* = 0.94 μV; Surface: *M* = −1.07 μV; see Fig. 2). The simulation, which was run with a first order autocorrelation of 0.97 at lag 1, identified any sequence of consecutive significant *t*-tests longer than 34 ms to be reliable.

## 4 Discussion

When 4-month-old infants visually observe a touch to another person’s hand, this modulates the feedforward stages of somatosensory processing of a concurrently presented vibrotactile stimulus. The 4-month-old infants tested in the current experiment, like adults (Bufalari et al., 2007), showed a reduction of the amplitude of the contralateral SEP during visual observation of touches to a hand as compared to visual observation of touches to a surface. This reduction may, as is thought to be the case in adults, reflect the interference of two simultaneous stimuli to the somatosensory cortex, i.e., the interference of the tactile stimulus to the personal hand with a signal coding the observation of touch to another person (Bufalari et al., 2007).

One alternative possible explanation of our findings is that when infants saw a hand (as opposed to a surface) being touched this led to a more widespread suppression across larger areas of the brain, rather than the more specific modulation of somatosensory processing which we hypothesised. For instance, viewing a hand being touched may have attracted visual attention, resulting in suppression outside visual areas. However, other aspects of our findings cast significant doubt on this interpretation. Firstly, the observed modulations of somatosensory processing by visual condition (hand vs. surface) occur early in somatosensory processing (by 92 ms). Thus, any modulations of somatosensory processing likely occur in S1 and/or S2 (Hari and Forss, 1999) as result of bottom-up features of multisensory stimulation rather than a top-down modulation of attention. A counter to this argument might be that the infants were able to anticipate whether the touch would occur on the hand or surface prior to the onset of the vibrotactile stimulus (which was timed to co-occur with the visual tactile event). However, this is extremely unlikely because: i) the trajectories of brush movement in the visual stimuli were designed to be very similar up until the point of the touch, and ii) although it is argued that infants encode and respond to action goals from 5 to 7 months (Csibra, 2008, Woodward, 1998), and that infants can predict whether actions have goals from around 9–12 months (e.g., Cannon and Woodward, 2012, Falck-Ytter et al., 2006, Southgate and Begus, 2013, Southgate et al., 2010), even adults show a very limited ability to predict the specific destination of an action based on its early trajectory (Naish et al., 2013). As such it is unlikely that the early stages of the visual trajectories of the paintbrush towards the hand or the surface led to a top down modulation of processing of the subsequent somatosensory stimulus, particularly given the limitations in visual acuity at 4 months of age necessary to form such precise action predictions (Dobson and Teller, 1978).

A second reason to discount an account of the observed SEP modulations in terms of a broad-based suppression of cortical activity is that the SEP modulations are specific to hemisphere: the centrally presented visual touch to the hand rather than to the surface leads to attenuation of the SEPs in the contralateral hemisphere. But for ipsilateral SEPs, a more positive deflection was found in the Hand than in the Surface condition (i.e., an enhancement when a touch to a hand is observed). It is possible that this significant effect of Hand vs. Surface in both hemispheres is due to the activation of somatosensory areas by the visually perceived tactile event, but whereas that is suppressed by the concurrent vibrotactile signal reaching somatosensory cortex contralateral to the presentation of the touch, this vibrotactile somatosensory input signal does not reach ipsilateral areas to achieve this suppression.

That seen touches to a body part can influence cortical signatures of the feedforward stages of somatosensory processing in 4-month-old infants is consistent with the assertion that vicarious mapping plays a foundational role in early social perceptual development (e.g., Marshall and Meltzoff, 2014). These findings also underscore the importance of tactile and multisensory tactile-visual processes in early social perception (see Bremner and Spence, 2017, Fairhurst et al., 2014, Filippetti et al., 2013) where multisensory interactions have typically been studied in the audiovisual domain. But what is the precise basis of the visual-tactile mapping that we have observed? One possibility is that infants by 4 months of age are sensitive to the highly socially-specific properties of the visual event specifying a touch occurring to an observed hand. However, given that we have only drawn the relatively crude comparison of a hand being touched vs. a surface being touched there is the potential that more low level aspects of the visual event served to modulate somatosensory processing in the fashion which we observed. We consider this next.

The Hand vs. Surface contrast was limited to one viewpoint of the hand, with only one type of touch action. We chose this simple contrast in order to maximise the potential of observing vicarious mapping in 4-month-olds given a number of known and potential constraints are operating at this age: i) poorer visual acuity (Dobson and Teller, 1978), ii) limitations in the ability to generalise object (and hand) representations across different viewpoints (Cohen and Younger, 1984), iii) less well elaborated visual representations of human bodies and their parts (see, e.g., Slaughter et al., 2004), and iv) the small number of trials (and thus conditions) which it is possible to present 4-month-old infants with in a single session. Our simple contrast was designed to overcome as many of these potential limitations as possible, and provide infants with visual information specifying tactile events in a canonical and unambiguous manner. However, it remains to be determined to what extent the observed effects generalise across a range of contexts. One concern here is that our effect might be specific to the stimuli which we have used due to irrelevant low level features of those stimuli rather than their vicarious tactile content. We think this is unlikely for two reasons: i) the only differences between the Hand and Surface conditions were whether or not the paintbrush was applied to the hand or the adjacent surface, and ii) the effects we found (suppression of early contralateral components of the SEP in the Hand condition) are very similar in nature to those observed in adult participants (Bufalari et al., 2007).

Further experimentation will not only permit more confidence in ruling out a low level explanation of the kind considered above. It will also be crucial for determining the specificity (or generality) of the visual-somatosensory effect which we have reported here in 4-month-olds. One question concerns whether infants will show the same visual influences on somatosensory processing across a range of different orientations of a hand (or other body part). One prediction might be that seeing a hand as if it is one’s own (in first person perspective) might lead to a greater vicarious mapping effect of the kind reported above, if only because infants are more likely to have experienced touches at the same time as seeing touches on a hand observed in that orientation (see later). This will of course depend on the degree to which infants generalise representations of their own vs. others’ hands across viewing angles, an aspect of body representation which may change significantly during infancy.

A further question concerns whether the effect we have observed is general across social and non-social stimuli. Further experiments in which effects of touches to a hand vs. touches to an inanimate object (e.g., a toy) are compared would help shed light on the extent to which the visual modulations of somatosensory processing we have observed are a specifically social crossmodal mapping process, or might also underlie the development of the crossmodal basis of representing or experiencing ownership over one’s own body (see Zeller et al., 2015). It is important to note however, that even if the effect we have observed is in fact a crossmodal mapping which generalises across social and non-social domains, it might nonetheless play an important role in driving the development of the vicarious mappings of touch which serve as social perceptual adaptations in the adult brain (see Tsakiris, 2017).

Current influential accounts of multisensory interactions in infancy argue for a key role for putative “amodal” (or redundant) properties of stimulation, such as common temporal onset, intensity or spatial location across the senses, in guiding young infants’ attention to the relevant properties of social and non-social events (see Bahrick and Lickliter, 2014). In the current study, whilst there is some temporal correspondence across the visual and tactile events across both conditions, the difference between conditions (i.e., whether or not the observed touch event occurred on a visually-specified hand) modulated somatosensory processing. This visual feature is arbitrarily (non-redundantly) related to the tactile information, which it modulates in 4-month-olds’ brains. Rather than being governed by “amodal” redundancy across the senses, it therefore appears that when observing touches to another person, infants’ are sensitive to more specific features of multisensory stimulation.

But how this kind of multisensory skill might have come about is still an open question. One possibility is that there is an experience independent predisposition to mirror vicarious tactile experiences to one’s own tactile perception in the service of sensory empathy (e.g., Marshall and Meltzoff, 2014). However, we should not rule out a potential role for experience and associative learning. Recent studies demonstrate the prevalence of hands (as well as faces) in the visual field in the early months of life (Fausey et al., 2016). Seeing hands being touched in the visual field (particularly when those hands are one’s own) is likely to be correlated with somatosensorially mediated touches. It is possible that such associations are learned early in the first year of life (by 4 months of age), and go on to underpin the development of more sophisticated and specific forms of sensory empathy. Such an account would be consistent with recent associative learning accounts of the development of action mirroring in early life (Cook et al., 2014, Heyes, 2010, Ray and Heyes, 2011).

In summary, the current findings show that at 4 months of age somatosensory processing is modulated by observing touches delivered to another person, and therefore that the infant somatosensory cortex is involved not only in the personal experience of touch but also supports vicarious mapping of others’ tactile experiences. Further studies will help clarify the precise basis of these visual-tactile interactions in early life.

## Conflict of Interest

None.
